# Effect of Long-Term Cryopreservation on the Stemness of Stem Cells of Apical Papilla

**DOI:** 10.1155/2022/6004350

**Published:** 2022-12-27

**Authors:** Anna Digka, Eleni Gounari, Kokkona Kouzi-Koliakou, Kleoniki Lyroudia

**Affiliations:** ^1^Laboratory of Endodontology, Dental School, Aristotle University of Thessaloniki, Thessaloniki, Greece; ^2^Laboratory of Histology and Embryology, Medical School, Aristotle University of Thessaloniki, Thessaloniki, Greece

## Abstract

Stem cells of apical papilla (SCAPs) are considered a subpopulation of dental stem cells with unique properties. They originate from a developing tissue, the apical papilla of developing teeth, a characteristic that enhances their stemness. Banking of these stem cells can offer a source of dental stem cells for future regenerative therapies. Until now, only the effect of six months' cryopreservation on SCAPs has been studied. In this study, the long-term (19 months) effect of cryopreservation on SCAPs was examined by means of estimation of their differentiation's capacity, flow cytometry immunophenotypical characterization, and molecular characterization of the main transcriptional factors that coincide with pluripotency. As was indicated from our results, 19-month cryopreservation of SCAPs did not affect negatively their stemness; since no significant difference was observed on their typical fibroblast-like morphology, they retained their differentiation capacity, and no discrepancies were found either on immunophenotypical level or molecular level.

## 1. Introduction

Regenerative endodontic procedures (REPs) are based on the use of the “holly triad,” stem cells (SCs), growth factors, and scaffolds [1]. Apical papilla comprises the main source of stem cells that are employed during REP, since, in most of these cases, dental pulp is necrotic, which means absence of dental pulp stem cells.

Stem cells of apical papilla (SCAPs) are considered a subpopulation of dental stem cells with unique properties [2]. The most striking characteristic of SCAPs is their origin, as they derive from a developing tissue, the apical papilla of the developing tooth. On the other hand, the rest of stem cells in the human body (somatic stem cells) originate from fully developed organs. The above characteristic of SCAPs enhances their stemness.

They are present only during the early stages of tooth development, since their residence, apical papilla, is resolved after the fulfillment of root development [3]. In that line, banking of SCAPs can offer a source of dental stem cells for future regenerative therapies. Until now, only the effect of six months' cryopreservation on SCAPs has been studied [4].

Therefore, in this study, the long-term effect of cryopreservation will be examined by means of estimation of their differentiation's capacity, flow cytometry immunophenotypical characterization, and molecular characterization of transcriptional factors of genes that coincide with pluripotency before and after 19 months cryopreservation.

## 2. Materials and Methods

The study was carried out at the Department of Endodontology, School of Dentistry, and the Department of Histology and Embryology, Medical School, Aristotle University of Thessaloniki, Greece, and was approved by the Ethical Committee of the School of Dentistry, Aristotle University of Thessaloniki (72/10-12-2019).

Healthy human immature third molars, extracted for orthodontic purposes, were collected from 16 to 22-year-old donors, after their or their parents' informed consent. All the donors had free medical history since certain diseases can alter the gene expression of the transcription factors. Prior to extraction, radiographic examination was performed for the verification of the presence of the apical papilla. The extractions were held under the most aseptic conditions and most gentle atraumatic manipulations for the preservation of apical papilla. The teeth were proceeded immediately for apical papillae' separation.

### 2.1. SCAP Isolation, Culture, and Proliferation

SCAPs were isolated and cultured as previously described by Sonoyama et al. [2]. Briefly, apical papilla was digested in a solution of 3 mg/ml collagenase type I and 4 mg/ml disposed for 30 min at 37°C, passed through a 70 mm strainer to obtain a single-cell suspension, and seeded into 25 cm^2^culture flasks containing alpha-modification of Eagle's medium supplemented with 15% fetal bovine serum, ascorbic acid 2-phosphate, glutamine penicillin, and streptomycin. Cultures were incubated at 37°C in a humidified atmosphere supplemented with 5% CO_2_. Medium was changed twice a week. Upon reaching 80% confluence, SCAPs were detached using 0.05% Trypsin-EDTA, reseeded, and cultured for further study.

### 2.2. Cryopreservation

Between 2-3 passage SCAPs were detached using 0.05% Trypsin-EDTA, centrifuged at 800*g* for 10 min, and resuspended in fetal bovine serum containing 10% DMSO in a cellular density of 3-4 *∗* 10^6^ cells per ml. Gradual freezing was performed, and the cells were finally stored in liquid nitrogen for 3, 8, and 19 months to test their characteristics upon thawing.

### 2.3. Before and After Cryopreservation Periods in Different Time Points (3, 8 and 19 Months), SCAPs Cultures were Studied for

#### 2.3.1. SCAP Differentiation Capacity

SCAPs were seeded onto 24-well plates at cellular concentration of 2 × 10^4^ per well. Aiming at the cell differentiation to test their osteogenic, adipogenic, and chondrogenic capacity, appropriate mediums were added in SCAP cultures for 28–32 days with medium changes every 2-3 days and incubation in 37°C with 5% CO_2_. After the end of induced differentiation, staining (Alizarin Red, Oil Red, and Alcian blue) was performed related to each differentiation.

#### 2.3.2. Immunophenotypical Characterization of SCAPs

Regarding the characterization of the SCAPs, flow cytometry (FCM) was performed for the detection of typical surface marker expression. In brief, upon detachment of cells with Trypsin-EDTA 1*x* in PBS and mild centrifugation, staining with monoclonal antibodies (mAbs) CD24, CD146, CD105, CD90, CD34, CD44, CD29, and CD45 was performed for 15 min in absence of light, and finally analysis in BD FACS Calibur (BD Biosciences) was followed.

#### 2.3.3. Molecular Characterization of SCAPs

Molecular characterization of SCAPs for NANOG, OCT4, CMYC, SOX2, KLF4, SALL4, ESRRB, ZNF217, and ZNF878 genes (Table 1) [5–16] was followed. Quantification of transcription factors genes' expression levels was performed via real-time PCR with the use KAPA SYBR® FAST one step qPCR Master Mix (2*X*) Kit. More precisely, SCAPS freshly isolated or after 3, 8, and 19 months in liquid nitrogen, were cultivated up to 4 passage and detached using 0.05% Trypsin-EDTA followed by RNA extraction procedure according to the manufacturer's instructions (Macherey Nagel, Düren, Germany). A subsequent RNA quantification in a NanoDrop ND-1000 UV-Vis Spectrophotometer was done. After a cautious primer design (Table 2) as well as HPLC Purification (Lab Supplies, Athens, Greece), Q-PCR for 10 ng of RNA template was performed, the results of which were analyzed by using ddCt algorithm for the analysis of the relative changes in gene expression. Rotor Gene 6000 genetic amplification detection system (Corbett Life Science) was used to perform the reaction. GAPDH was used as house-keeping gene for the normalization of gene expression.

### 2.4. Statistical Analysis

Data were presented as mean ± SD, and Student's *t*-test (unpaired, two-tailed) was used for the two-group comparisons. Differences were considered statistically significant at a value of *p* ≤ 0.05.

## 3. Results

### 3.1. Before Cryopreservation

**Figure 1:**
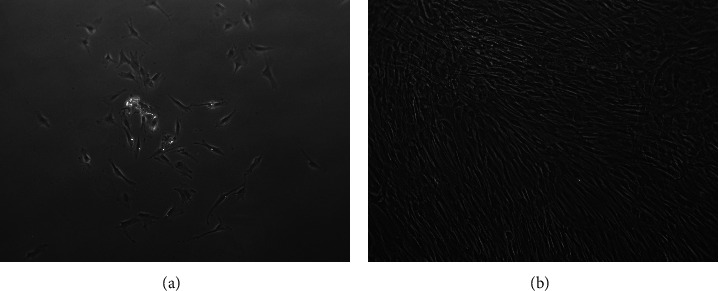
Isolation and culture of SCAPs. First attachment at fourth day (a) and full confluency at eighteenth day (b).

SCAPs were isolated according to the Sonoyama et al. [2] method. First attachment of the stem cells on plastic surfaces was observed 1 to 4 days after cells' plating. SCAPs showed the typical fibroblast-like morphology after attachment while their expansion rate was gradually augmented (Figure 1). The cultures were ready for further manipulation between the third and fourth week of incubation (3.5 × 10^6^ cells).

**Figure 2:**
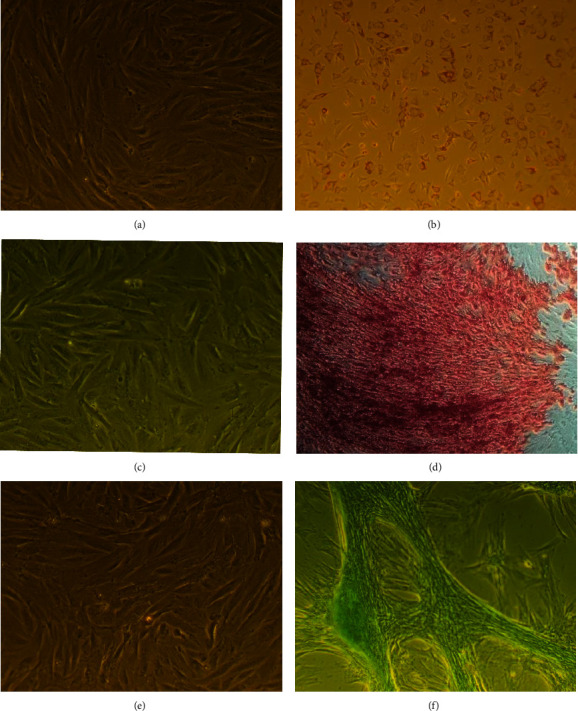
Representative microscopic images from induced three-layer differentiation of SCAPs. Induced adipogenic differentiation: control (a) and staining with Oil Red (b). Induced osteogenic differentiation: control (c) and staining with Alizarin Red (d). Induced chondrogenic differentiation: control (e) and staining with Alcian blue (f).

The induced differentiation of SCAPs confirmed osteogenic, adipogenic, and chondrogenic capacity as confirmed with positive Alizarin Red, Oil Red, and Alcian blue staining, respectively, and in comparison, with the control unstained groups (Figure 2).

**Figure 3:**
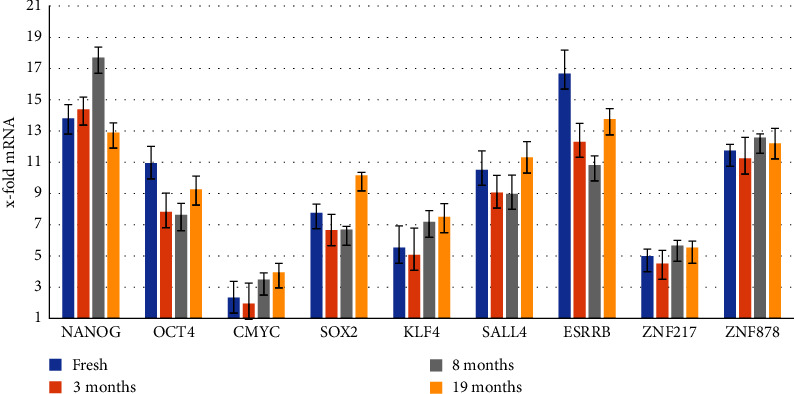
Immunophenotypic characterization of SCAPs before and after 3, 8, and 19 months of freezing reveals that no significant differences present at surface markers expression percentages.

Flow cytometric results regarding surface marker expression revealed that SCAPs were highly positive for CD24, CD29, CD44, CD90, CD105, and CD146 and negative for CD34 and CD45. Specifically, the ranging expression of CD24 was 98.70%, of CD29 94.2%, of CD44, 92.00%, of CD90, 99.2%, of CD105, 98.65%, of CD146, 97.44%, of CD34, 0.94% and of CD45 0.79% (Figure 3).

**Figure 4:**
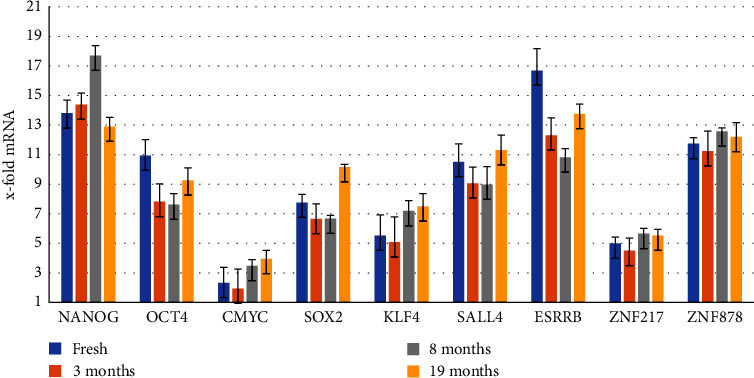
Analysis of the molecular profile of SCAPs before and after freezing reveals that no changes occur even after 19 months in liquid nitrogen.

In the transcriptional level, as proved by Q-PCR, most transcription factor-related genes are highly expressed especially in case of NANOG, OCT4, ESRRB, and ZNF878 genes where 14, 11, 17, and 12-fold augmentation was observed (Figure 4).

### 3.2. After Cryopreservation

**Figure 5:**
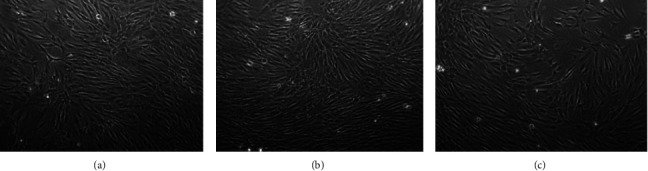
Depiction of the morphology of 3 (a), 8 (b), and 19 (c) months-frozen SCAPs upon thawing shows that no significant difference is observed on their typical fibroblast-like morphology in comparison with freshly isolated and cultivated SCAPs.

After 3, 8, and 19 months of cryopreservation and thawing of SCAPs, their first attachment on plastic surfaces was observed 2 to 6 days after cells' plating. SCAPs retained the typical fibroblast-like morphology after attachment while their expansion rate was gradually augmented as in the primary cultures (Figure 5). The cultures were ready for further manipulation between the third and fourth week of incubation (3.5 × 10^6^ cells).

SCAPs retained their differentiation capacity after 3, 8, and 19 months of cryopreservation as it was confirmed by induced differentiation of SCAPs to osteogenic, adipogenic, and chondrogenic lineage positive staining with Alizarin Red, Oil Red, and Alcian blue, respectively, and in comparison, with the control unstained groups (data not shown).

Also, the SCAPs retained their immunophenotypic characteristics after 3, 8, and 19 months of freezing as it was shown by flow cytometry. More specifically, flow cytometric results regarding surface marker expression revealed that SCAPs were highly positive for CD24, CD29, CD44, CD90, CD105, and CD146 and negative for CD34 and CD45: the ranging expressions after 3, 8, and 19 months of cryopreservation of CD24 were 97.70%, 94.20% and 94.23%, of CD29 92.00%, 91.50% and 90.00%, of CD44, 94.20%, 94.00% and 92.00%, of CD90, 96.60%, 89.60% and 96.70%, of CD105, 95.00%, 97.20% and 92.10%, of CD146, 98.10%, 95.30% and 92.80%, of CD34, 0.94%, 0.92% and 1.30% and of CD45 1.12%, 0.90% and 1.30% respectively (Figure 3).

Furthermore, cryopreservation did not affect the SCAPs in transcriptional level, as depicted by Q-PCR: the majority of transcription factor-related genes were highly expressed in SCAPs after 3, 8, and 19 months of cryopreservation; also, NANOG, OCT4, ESRRB, and ZNF878 genes maintained their high augmentation (Figure 4).

The above results indicate that 19-month cryopreservation did not affect negatively the stemness of SCAPs; since no significant difference was observed on their typical fibroblast-like morphology, they retained their differentiation capacity, and no discrepancies were found either on immunophenotypical level or molecular level.

## 4. Discussion

Stem cells of apical papilla (SCAPs) have recently gained great attention due to their implication in regenerative endodontic procedures (REPs). They are considered the “apical papilla treasure” [17]. Along with dental pulp stem cells (DPSCs) (18), they consist of the most representative dental stem cells. Nevertheless, their superiority over DPSCs is well established [17, 18]. SCAPs exhibit faster colony-forming and greater deposition of mineralized tissue matrix than DPSC [19]. Furthermore, SCAPs can survive the pulpal and periapical inflammation, due to the collateral vascularization of the apical papilla [20], in contrast to DPSC, that are obscured in necrotic dental pulps, and thus, unobtainable for REPs. They, also, appear the ability of proliferation in large numbers in vitro [21].

The immunophenotypical characterization by FCM has shown that the isolated primary cell populations comprised the characteristics of SCAPs. Pluripotency marker CD24 is considered SCAP specific, since it cannot be detected in other mesenchymal stem cells (MSCs), including DPSCs [22]. In this study, the cell populations were highly positive for CD24 (expression: 98.70%). According to Liu et al. [22], to evaluate the pluripotency, high positivity to CD24 is a perquisite for the selection of cell populations. They were, also, highly positive for CD146 (97.44%) that indicates their perivascular location [22] as well as highly positive for the typical MSC markers CD90 (99.2%), CD105 (98.65%), CD29 (94.20%), and CD44 (92.00%), indicating their mesenchymal origin [22]. Finally, they were negative for the leukocyte precursor marker CD45 (0.79%) and hematopoietic marker CD34 (0.94%). This almost lack of expression for CD45 and CD34 confirms firstly their stromal origin and secondly the purity of cell cultures, without contamination with hematopoietic precursors [2, 23].

The abovementioned immunophenotypic characteristics of SCPAs were preserved after 19 months cryopreservation, as it was shown from our results, indicating safe cryopreservation of SCAPs.

The morphological differentiation of SCAPS to osteocytes, adipocytes, and chondrocytes before and after cryopreservation also confirms that cryopreservation did not affect negatively their stemness capacity and ability to be differentiated towards the three germ layers successfully, as it was shown after the appropriate staining of induced differentiated cultures.

In this study, the expression of genes that coincide with pluripotency has been evaluated in SCAPs for the first time, before and after cryopreservation, to characterize in detail the molecular profile of this cell subpopulation and study the effect of cryopreservation on their molecular profile.

From our results, SCAPs express in different *x* fold amount NANOG, OCT4, C-MYC, SOX2, KLF4, SALL4, ESRRB, and ZNF217 genes responsible for differentiation and reprogramming of cells which are commonly expressed at high levels in embryonic (ESC) and mesenchymal stem cells (MSC). Although most transcription factor-related genes, like NANOG, OCT4, and ZNF878, are highly expressed, the ESSRB gene expression is remarkably high reaching 17 *x*-fold augmentation.

ESSRB is a crucial determinant for the maintenance of pluripotency since its depletion or removal leads to differentiation. Regarding self-renewal, ESSRB can functionally substitute NANOG: in the absence of GSK3I and LIF, NANOG cannot preserve self-renewal while ESSRB has this capacity [11]. Although ESSRB can rescue self-renewal in the absence of NANOG, with simultaneous presence, they can express enhanced efficiency on self-renewal. Synergetic action of ESSRB with NANOG induces the pluripotency of differentiated cells and stabilizes ESC self-renewal through positive feedback [12].

Additionally, ESSRB promotes reprogramming of somatic cells to pluripotent state, even in the absence of NANOG [12], and has the unique capacity to reprogram NANOG^−/−^ cells to naïve pluripotency. ESSRB is considered as a marker of reprogramming progression in somatic cells and along with Nanog can substitute SOX2 while OCT4 remains needful for vigorous reprogramming [13].

ESSRB expression provides stability to the network of naïve pluripotency, and ESSRB positive cells are considered to represent an “elite” subpopulation [13]. Therefore, SCAPs, as indicated from our results, can be numbered among the “elite.” Human pluripotent stem cells in naïve state highly express, among other genes, ESSRB, NANOG, OCT4, SOX2, and KLF4 [14]. As shown from our results, SCAPs highly express Essrb, Nanog, Oct4 and in a lower grade SOX2 and KLF4, so it could be considered “naïve-like” stem cells.

The overexpression of ESRRB gene in SCAPs, along with the high expression of NANOG, OCT4, and ZNF878 genes, as found in our study, confirms their undifferentiated state and rationalizes their high pluripotency potentiality. Additionally, to their capability for dentinogenic differentiation [2], SCAPs exhibit high osteogenic potential [19]. Furthermore, they have proangiogenic and angiogenic properties and can promote the vascularization [23] as well as they demonstrate neurogenic [24], adipogenic [25], chondrogenic [26], and hepatogenic [27] differentiation capacity.

Our results are in agreement with the results of Bakopoulou et al. [28, 29] and Wu et al. [30] regarding the expression of NANOG, and OCT4, while regarding the rest of the genes examined in this study, no other data exist for their expression in SCAPs.

Furthermore, it also evident by this study that cryopreservation did not affect the molecular profile of SCAPs, since they continued to express the abovementioned genes in almost the same levels, as in primary cultures.

SCAP-based regeneration dental therapies include regenerative endodontic procedures whose main goal is the fulfillment of root development in immature permanent necrotic teeth and cell-based therapies in combination with minimal access flap surgery for periodontal reconstruction [31–34]. Besides dentin-pulp and periodontal regeneration, SCAP-based therapeutic applications are implicated in bio-root engineering, in the regeneration and repair of neural tissue, and in ischemic diseases even in immunotherapies [35]. New data in the translational research regarding SCAPs are shown in our study that further elucidate and rationalize their pluripotency capacity.

Our results, on the study of cryopreservation of SCAPs, agree with the results of Ding et al. [4], where six-month freezing was studied.

## 5. Conclusions

Stem cells of apical papilla (SCAPs) are obtained from extracted human immature third molars or immature teeth extracted for orthodontic purposes during routine dental procedures, which are less invasive compared with the harvesting processes required for other types of stem cells, e.g., bone marrow stem cells. This high accessibility, in combination with their multipotential differentiation capacity along with their undifferentiated state, and their ability for long-term safe cryopreservation and banking, as indicated from our results, highlight SCAPs as excellent candidates for regeneration procedures in general. Further studies, with longer banking periods are necessary, to study the effect of cryopreservation on SCAPs.

## Figures and Tables

**Table 1:** Genes' properties (main properties of genes that coincide with pluripotency) (ESC: embryonic stem cells).

Gene	Main properties
NANOG	(i) Regulation of the differentiation and the pluripotency (ii) Sustaining the pluripotency of epiblast and impeding it from differentiation (iii) Induction of self-renewal and the preservation of pluripotency (5)

OCT4	(i) Yamanaka factor, essential for the generation of pluripotent stem cells of fibroblasts (ii) Regulation of the pluripotency and differentiation of ESC (6)

CMYC	(i) Yamanaka factor, essential for the generation of pluripotent stem cells of fibroblasts (6) (ii) Regulation of the pluripotency and differentiation of ESC (6) (iii) Regulation of cell cycle entry and apoptosis (7) (iv) Promotion of cell growth proliferation (7) (v) Suppression of terminal differentiation (7)

SOX2	(i) Yamanaka factor, essential for the generation of pluripotent stem cells of fibroblasts (6) (ii) Regulation of the pluripotency and differentiation of ESC (6) (iii) Contributes to the development and homeostasis of adult tissues (8)

KLF4	(i) Yamanaka factor, essential for the generation of pluripotent stem cells of fibroblasts (6) (ii) Regulation of the pluripotency and differentiation of ESC (6) (iii) Regulation of cell apoptosis, proliferation, and differentiation (9)

SALL4	(i) Preservation of the stemness of ESC (ii) Regulation of somatic cells reprogramming to pluripotency (10).

ESRRB	(i) Controls ESC self-renewal and fosters their reprogramming (11) (ii) Maintenance of pluripotency (11) (iii) Can functionally substitute Nanog regarding self-renewal (11) (iv) Promotes reprogramming of somatic cells to pluripotent cells (12) (v) Provides stability to the network of naïve pluripotency (13, 14)

ZNF217	(i) Orchestrating the tissue development and differentiation (ii) Epigenetic regulation in the expression of NANOG, OCT4, and SOX2 (iii) Decreased expression in differentiated cells (15)

ZNF878	(i) Orchestrating the tissue development and differentiation (ii) Regulation of transcription (16)

**Table 2:** Primer design for Q-RTPCR (forward 5′-3 and reversed 5′-3′ sequences for molecular analysis).

Gene	Forward 5′-3′	Reversed 5′-3′
ZNF217	GGAATGGAACAACAGCGG	AAACATGCCAACTCAATCCCT
ZNF878	GAAATAAATCCCTTACCACA	ACCCTATGAGTGTACACAAT
ESRRB	CCCACTTTGAGGCATTTCAT	ACATTGCCTCTGGCTACCAC
SALL4	AGTACAGCTCCGGAGAAGTC	AATGCTGTGCGGAGTTCTTC
KLF4	CCGCCAGCGGTTATTCGGGG	GCAGCCACCTGGCGAGTCTG
SOX2	CGAGCTGGTCATGGAGTTGTA	TACAGCATGATGCAGGACCA
NANOG	TGCTGGAGGCTGAGGTATTTCTGTCTC	AGTCCCAAAGGCAAACAACCCACTTC
OCT4	TGGGACTCCTCCGGGTTTTG	AGCCCTCATTTCACCAGGCC
CMYC	GCACAAGAGTTCCGTAGCTG	AAAGGCCCCCAAGGTAGTTA
GAPDH	ACGGCAGGTCAGGTCCACCA	ACTGTGGATGGCCCCTCCGG

## Data Availability

The data supporting the conclusions of the study are available from the corresponding author upon request.
